# Acute Hypertensive Retinochoroidopathy Secondary to an Anti-cancer Drug (apatinib): The First Case Report

**DOI:** 10.3389/fmed.2021.677941

**Published:** 2021-06-23

**Authors:** Bangtao Yao, Gang Liu, Bei Wang, Qian Cao

**Affiliations:** ^1^Department of Ophthalmology, Lishui District People's Hospital, Nanjing, China; ^2^Department of Ophthalmology, General Hospital of Eastern Theater Command, Nanjing, China

**Keywords:** hypertensive retinochoroidopathy, apatinib, BRB, elschnig spots, RPE, SRD

## Abstract

**Background:** Acute hypertensive retinochoroidopathy is a rare, severe ocular disease, characterized by retinal and choroidal ischaemia. Untreated cases are associated with high mortality and poor visual outcomes. Patients subjected to treatment with the anti-neoplasic drug apatinib may trigger this disease. The purpose of this article is to describe in detail an acute hypertensive retinochoroidopathy in a young Chinese woman treated with apatinib.

**Case Presentation:** A 40-year-old young Chinese woman presented a sudden but painless reduction of visual acuity in both eyes. She was previously diagnosed with gastric cancer and metastatic ovarian adenocarcinoma. The treatment consisted radical gastrectomy, transabdominal hysterectomy, bilateral adnexectomy, and 250 mg oral apatinib per day. After 58 days of apatinib administration, the patient immediately sought consult for a sudden decrease in vision. Her blood pressure was 208/136 mmHg and, based on the clinical manifestations, the patient was diagnosed with acute hypertensive retinochoroidopathy.

**Conclusions:** This is the first case report of an apatinib-related acute hypertensive retinochoroidopathy diagnosed using fundal photograph, fundus fluorescein angiography, and spectral-domain optical coherence tomography simultaneously. It is crucial to develop a suitable strategy for management and prevention of this adverse event.

## Introduction

Hypertension is a risk factor for several systemic conditions that results in severe morbidity and mortality. It is also a risk indicator of vision-threatening ocular diseases, including hypertensive retinopathy, choroidopathy, and neuropathy (1, 2). Malignant hypertension is the most severe form of hypertension, clinically defined as diastolic blood pressure above 130 mmHg. Almost 80% of untreated patients with the condition will die within 2 years (2). The combination of hypertensive retinopathy and choroidopathy, termed hypertensive retinochoroidopathy, is a rare and severe ocular condition that results in a sharp visual reduction due to malignant hypertension (3, 4).

Apatinib is a highly selective vascular endothelial growth factor receptor-2 (VEGFR-2) inhibitor, which has been proven effective and safe for cancer patients (5–7). However, several side effects have been reported such as hypertension, abnormal liver function, haematologic toxicity, and proteinuria (7, 8). Malignant hypertension is a rare complication of apatinib treatment, and drug-related acute hypertensive retinochoroidopathy has rarely been reported. In this work, we report a case of a 40-year-old Chinese woman diagnosed with acute hypertensive retinochoroidopathy after treatment with apatinib via simultaneous fundus photography, fundus fluorescence angiography (FFA), and spectral-domain optical coherence tomography (SD-OCT).

## Case Description

A 40-year-old Chinese woman presented a sudden painless reduction of visual acuity in both eyes. The patient had no history of high blood pressure, diabetes mellitus, chronic renal impairment, preeclampsia, renal artery stenosis, pheochromocytoma, or heart disease. In March 2019, she was diagnosed with gastric cancer and treated with radical gastrectomy and oral tegafur (40 mg twice a day). After the operation, the epigastric pain due to gastric cancer was relieved obviously, and her blood pressure remained normal during the follow-up period. Thirteen months later, she was diagnosed with ovarian metastasis of gastric cancer. She was treated with transabdominal hysterectomy, bilateral adnexectomy, 40 mg oral tegafur twice per day, and 250 mg apatinib daily. On the 58th day of drug administration, the patient immediately sought consult for a sudden decrease in vision. Her blood pressure was 208/136 mmHg while laboratory examination results showed that the liver, thyroid, and renal function remained normal.

**Figure 1:**
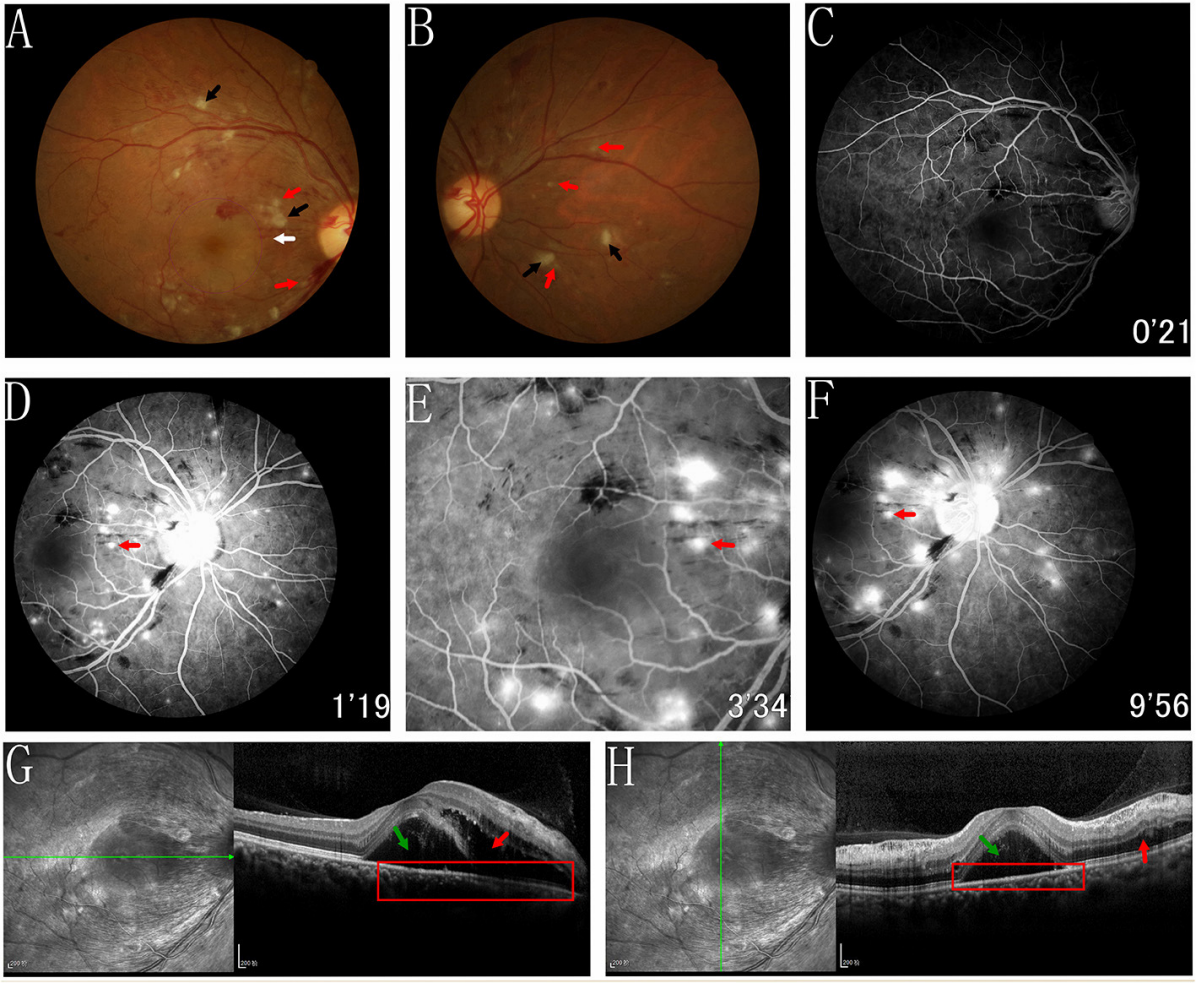
Fundus photograph, FFA and SD-OCT of the right eye. **(A,B)** Funduscopic examination revealed retinal edema, intraretinal hemorrhage, elschnig spots (white arrow and red arrows), hard exudates and cotton-wool spots (black arrows) in right eye. **(C)** FFA showed the delayed choroidal filling regarding areas of capillary nonperfusion. **(C–F)** FFA showed multifocal sites of patchy hyperfluorescence in the early phase, with leakage during the entire imaging process describing elschnig spots, and blockage from the cotton wool spots. **(G,H)** SD-OCT showed the IRF (red arrows), SRD (green arrows) with multilayered hyperreflective lesions indicating fibrinous materials, swelling of RNFL, and thinner and disorganisation of the EZ and RPE (red rectangles).

**Figure 2:**
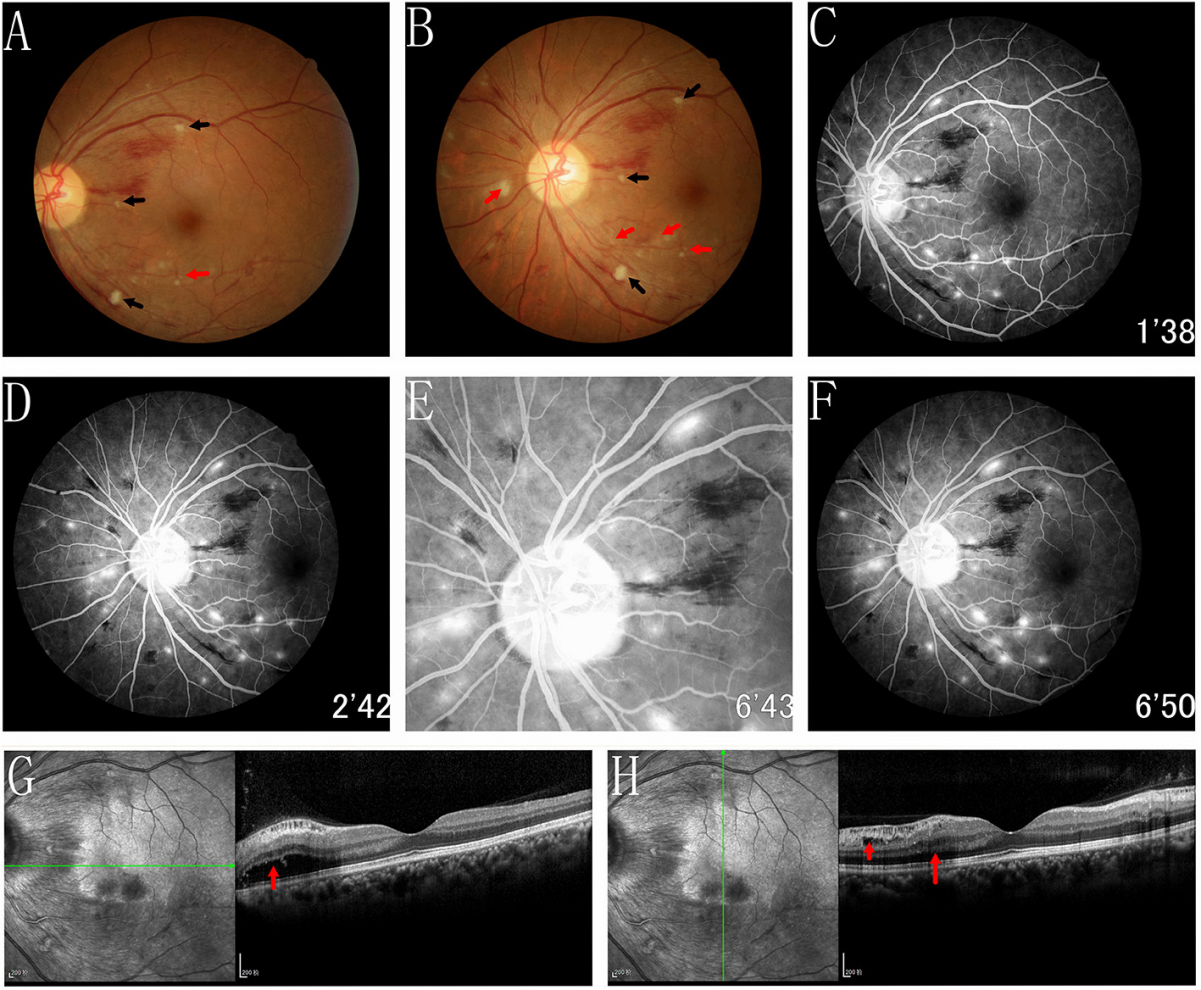
Fundus photograph, FFA and SD-OCT of the left eye. **(A,B)** Funduscopic examination revealed retinal oedema, intraretinal haemorrhage, elschnig spots (red arrows), hard exudates and cotton-wool spots (black arrows) in left eye. **(C–F)** FFA showed multifocal sites of patchy hyperfluorescence in the early phase, with leakage during the entire imaging process describing elschnig spots, and blockage from the cotton wool spots. **(G,H)** SD-OCT showed the IRF (red arrows) with multilayered hyperreflective lesions, swelling of RNFL and relatively preservation of the EZ and RPE were observed.

The best-corrected visual acuity (BCVA) was 20/80 OD and 20/50 OS. The anterior segment was normal. The pupils were equal, round, and reactive to light with no afferent pupillary defect. The intraocular pressure was normal in both eyes. Funduscopic examination revealed retinal oedema, intraretinal hemorrhage, elschnig spots, hard exudates, and cotton-wool spots in both eyes (Figures 1, 2A,B). FFA showed delayed choroidal filling in areas of capillary nonperfusion (Figure 1C). FFA also showed multifocal sites of patchy hyperfluorescence in the early phase, with leakage during the entire imaging process describing elschnig spots, and blockage from the cotton-wool spots (Figures 1, 2C–F). Spectral-domain optical coherence tomographic (SD-OCT) showed intraretinal fluid (IRF), serous retinal detachment (SRD) with multilayered hyperreflective lesions indicating fibrinous materials, swelling of the retinal nerve fiber layer (RNFL), disorganization of the ellipsoid zone (EZ), and retinal pigment epithelium (RPE) in the right eye (Figures 1G,H). IRF with multilayered hyperreflective lesions, RNFL swelling, and relatively preserved EZ and RPE were observed in the left eye (Figures 2G,H). Based on these findings, the patient was diagnosed with acute hypertensive retinochoroidopathy.

The patient was treated with sublingual 25 mg captopril, oxygenated in supine position, an electrocardiogram was performed immediately and her blood pressure monitored. After the hypertension symptoms decreased, 5mg amlodipine besylate was administrated daily and her blood pressure continued to be closely monitored. Oral tegafur and apatinib were maintained.

**Figure 3:**
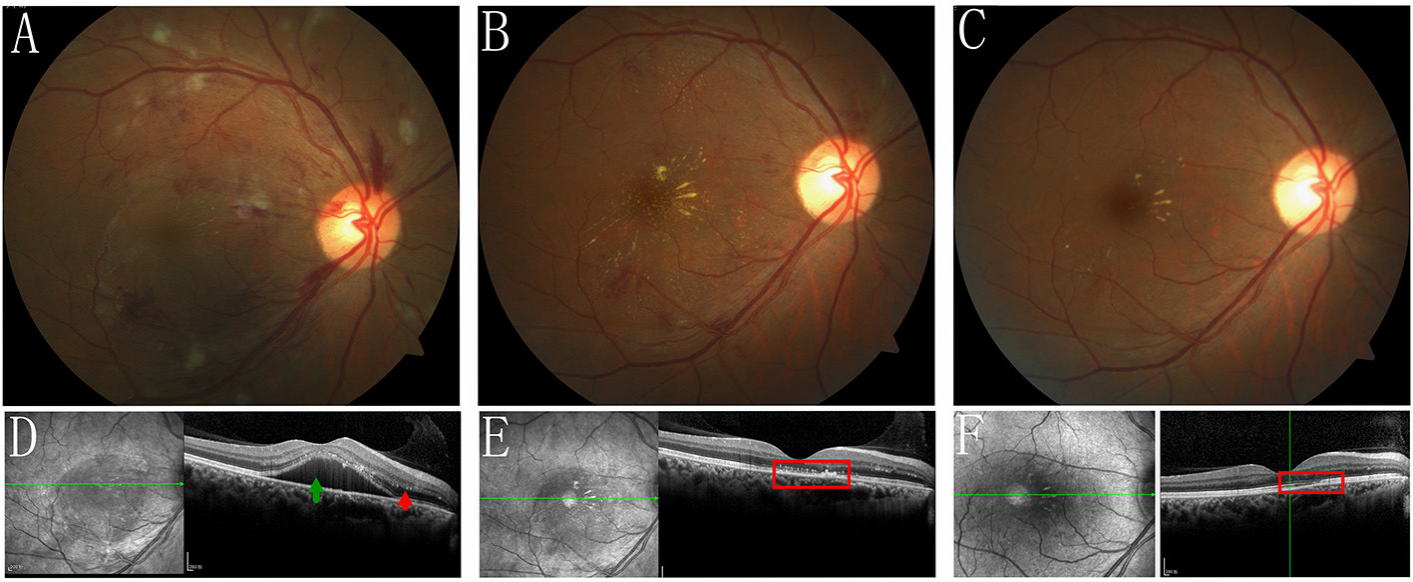
Fundus photograph, and SD-OCT of the right eye during the follow-up. **(A,D)** 1 week follow-up, the IRF (red arrow) and SRD (green arrow) were reduced remarkably. **(B,E)** 1 month follow-up showed that the IRF and SRD were entirely resolved, however, the unusually punctate hyperreflection foci on the RPE level were still observed in the right eye's macula by SD-OCT (red rectangle), indicating a previous elschnig spot (white arrow in Figure 1A and red arrows in Figures 1D–F) and stellate exudates were observed in the right eye's macula. **(C,F)** At the most recent follow-up, after 4 months, the fundus condition was further improved, however, interruption and disorganisation of the EZ and RPE on SD-OCT (red rectangles) and hard exudates were observed in the right eye's macula.

**Figure 4:**
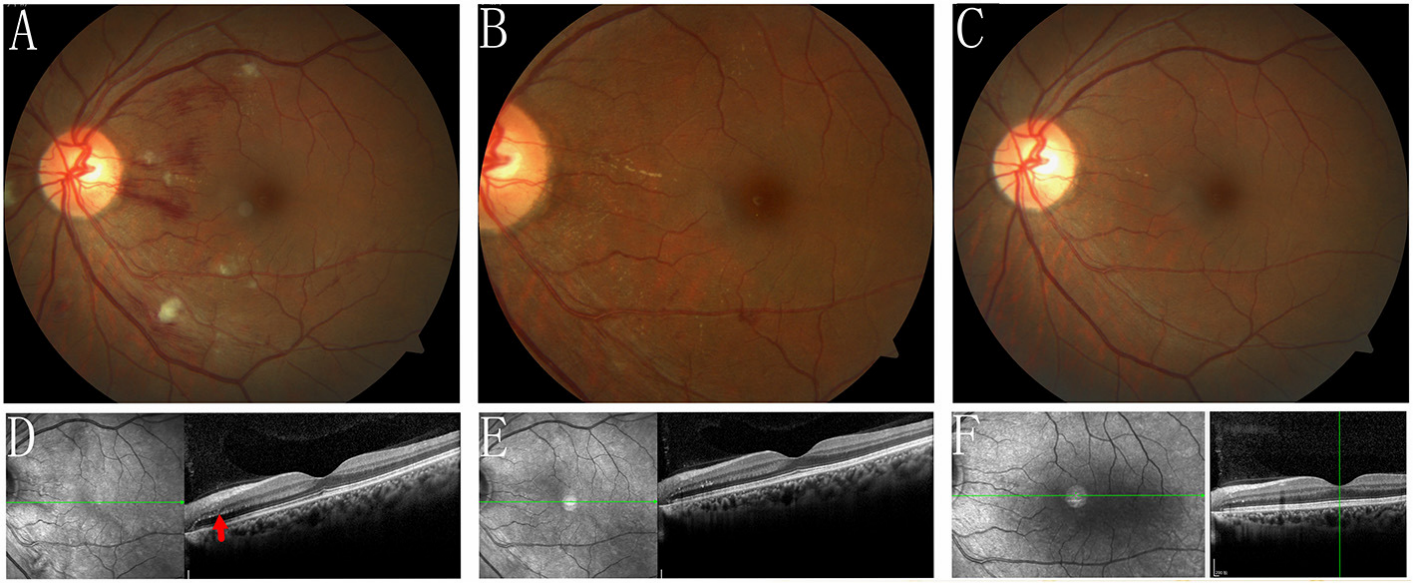
Fundus photograph, and SD-OCT of the left eye during the follow-up. **(A,D)** 1 week follow-up, the IRF (red arrow) and SRD were reduced remarkably. **(B,E)** 1 month follow-up showed that the IRF and SRD were completely resolved. **(C,F)** At the most recent follow-up, after 4 months, the fundus appeared basically normal.

One week later, IRF and SRD were significantly reduced in both eyes (Figures 3, 4A,D) and the BCVA improved to 20/50 OD and 20/30 OS.

During the 1-month follow-up period, the IRF and SRD were entirely resolved (Figures 3, 4B,E). However, the unusually punctate hyperreflection foci on the RPE level were still observed in the right eye's macula by SD-OCT, indicating a previous elschnig spot (shown on fundus photography and FFA) and stellate exudates were observed in the right eye's macula (Figures 3B,E). The BCVA improved to 20/60 OD and 20/40 OS.

Four months later, during her most recent follow-up, the patient showed improvement of the fundus condition (Figures 3, 4C). However, interruption and disorganization of the EZ and RPE on SD-OCT and hard exudates were observed in the right eye's macula (Figures 3C,F). The fundus appeared normal in the left eye (Figures 4C,F) and the BCVA then improved to 20/20 in both eyes.

Even though the patient developed alopecia, her blood pressure remained stable during the entire follow-up period.

## Discussion

Acute hypertensive retinochoroidopathy is a very rare and severe ocular disease that results in a sharp decrease in vision associated to malignant hypertension and commonly occurring in young patients (1). Malignant hypertension is the most severe form of hypertension associated with preeclampsia, renal artery stenosis, and pheochromocytoma (3).

Apatinib is a novel, oral, small-molecule, highly selective VEGFR-2 inhibitor, which has been proven to be well-tolerated and effective for gastric cancer patients (5–7). However, hypertension, abnormal liver function, haematological toxicity, and proteinuria have been reported as frequent adverse events (7, 8). Yang et al. (6) found that patients with drug-related hypertension had significantly longer survival than those without the disease and concluded this was a predictor of apatinib efficacy. Wei Chen et al. (7) recommended an initial dose of 250 mg apatinib daily as acceptable and safe for ovarian cancer patients.

In our case, the patient was treated with both oral apatinib and tegafur. Tegafur induces digestive symptoms as relatively common side effects, but hypertension is rarely observed (9). Taking into consideration the lack of hypertensive events during the patients' previous tegafur monotherapy, we concluded that the malignant hypertension and secondary acute hypertensive retinochoroidopathy were caused by apatinib. Based on the data, we suggest this be included as a Common Terminology Criteria for Adverse Events grade 3–4.

The referred case manifested the retinal oedema, intraretinal hemorrhage, cotton-wool spots, hard exudates, and elschnig spots in the fundus photography, consistent with previous cases (1, 3, 4). Retinal oedema and elschnig spots were clear signs of hypertensive choroidopathy. However, retinal arteriosclerosis and arteriovenous nicking were not evident in our case, and we concluded it was in the acute stage.

FFA is a useful modality for diagnosing hypertensive retinochoroidopathy (1). FFA of the intraretinal hemorrhage showed blocked fluorescence. Moreover, cotton-wool and elschnig spots appeared similar but significantly different in their representations (6). Elschnig spots appeared as different yellowish lesions at the RPE level, which represented the poor perfusion of the overlying choriocapillaris and reflected the ischaemic infarcts of the RPE and choroid (4). The cotton-wool spots represented the micro-infarction of the RNFL due to the occlusion of retinal arterioles (10). Compared to cotton-wool spots, elschnig spots were located deeper. Elschnig spots demonstrated the patchy hyperfluorescence in the corresponding choroidal ischaemic areas during the early and late-phase subretinal leakage. Cotton-wool spots revealed the hypofluorescence in the early phase, and it remained stable throughout the whole process. In addition, FFA showed delayed choroidal filling in areas of capillary nonperfusion.

When performing SD-OCT, the right eye revealed SRD, IRF with multilayered hyperreflective lesions, RNFL swelling, and disorganization of the EZ and RPE. In the left eye, IRF with multilayered hyperreflective lesions and swelling of the RNFL were observed. Retinal ischaemia can result in RNFL swelling. Additionally, it is well-known that choroidal ischaemia and secondary necrotic RPE can lead to blood–retinal barrier (BRB) disruption, allowing fluid leakage from the subretina and SRD formation (11). Consequently, we believe that IRF could be attributed to the breakdown of the internal BRB secondary to retinal capillary ischaemia.^4^ Fibrinous materials appearing as multilayered hyperreflective lesions on SD-OCT are also due to fibrinoid necrosis of choroidal arterioles and secondary BRB damage. In the present case, SRD and IRF occurred in the right eye due to the destruction of both internal and external BRB. However, the damage to the left eye was slight: the RPE and external BRB seemed intact, which manifested as IRF without SRD.

Fortunately, SRD, fibrinous materials, IRF, intraretinal hemorrhage, elschnig spots, hard exudates, and cotton-wool spots resolved rapidly in our case after timely antihypertensive treatment. The characteristic stellate hard exudates were visualized in the macula, and the BCVA remarkably improved, suggesting that reperfusion of the retinal capillaries and choriocapillaris promoted absorption. During the 1-month follow-up period, the punctate hyperreflection foci was still visible on the RPE level and could be explained by the residual deposition of subretinal fibrin after the absorption of subretinal effusion in the remission stage. The interrupted and disorganized RPE revealed that part of the RPE function was already irreversible in the right eye. This documented the severity of the breakdown of the external BRB.

Siegrist streaks are rare linear atrophic stripes that develop over choroidal arteries in hypertensive choroidopathy, recognized as indicators of poor prognosis for patients (12). Luckily, they were not present in this case.

It was decided to continue on the initial 250 mg apatinib daily dose suggested by oncologists since the advantages overweighed the disadvantages for the present patient. The treatment was recommended as safe for patients with ovarian cancer and proven to be effective during the follow-up period. Furthermore, the elevated blood pressure was controlled by a single antihypertensive drug (5 mg amlodipine besylate daily).

When dealing with this scenario, several noteworthy differential diagnoses should be carefully addressed such as diabetic retinopathy, radiation retinopathy, anemia, ocular ischaemic syndrome, and retinal vein occlusion. Clinical manifestations and acute elevated blood pressure can be useful to distinguish hypertensive retinochoroidopathy from the aforementioned diseases.

We understand this study has two main limitations: the sample size was small and indocyanine green angiography and autofluorescence were not performed as it helps in better understanding of the disease.

In conclusion, we presented a rare case of malignant hypertension and secondary acute hypertensive retinochoroidopathy related to apatinib. This study highlights the importance of physicians' full attention to hypertension and related complications in cancer patients treated with apatinib. Blood pressure should be closely monitored and cooperation between physicians and ophthalmologists should be established to ensure the appropriate treatment for the general patients' health.

Thus, it is crucial to develop suitable management and prevention strategies for these serious adverse events, but prompt diagnosis and immediate intervention are first and foremost.

## Data Availability Statement

The original contributions presented in the study are included in the article/Supplementary Material, further inquiries can be directed to the corresponding author.

## Ethics Statement

Written informed consent was obtained from the individuals for the publication of any potentially identifiable images or data included in this article.

## Author Contributions

BY wrote the manuscript, established the diagnosis, and reviewed the manuscript. GL edited the manuscript. BW consulted literatures. QC dealt with the figures. All authors contributed to the article and approved the submitted version.

## Conflict of Interest

The authors declare that the research was conducted in the absence of any commercial or financial relationships that could be construed as a potential conflict of interest.

## Abbreviations

BCVA: best-corrected visual acuity
BRB: blood retinal barrier
EZ: ellipsoid zone
FFA: Fundus fluorescence angiography
IRF: intraretinal fluid
RNFL: retinal nerve fiber layer
RPE: retinal pigment epithelium
SD-OCT: spectral-domain optical coherence tomographic
SRD: serous retinal detachment
VEGF: endothelial growth factor receptor.
