# Supplementary figures and images for: Acute Hypertensive Retinochoroidopathy Secondary to an Anti-cancer Drug (apatinib): The First Case Report
Source: Front Med (Lausanne). 2021 Jun 23;8:677941. doi: 10.3389/fmed.2021.677941 (PMC8260843; doi:10.3389/fmed.2021.677941)

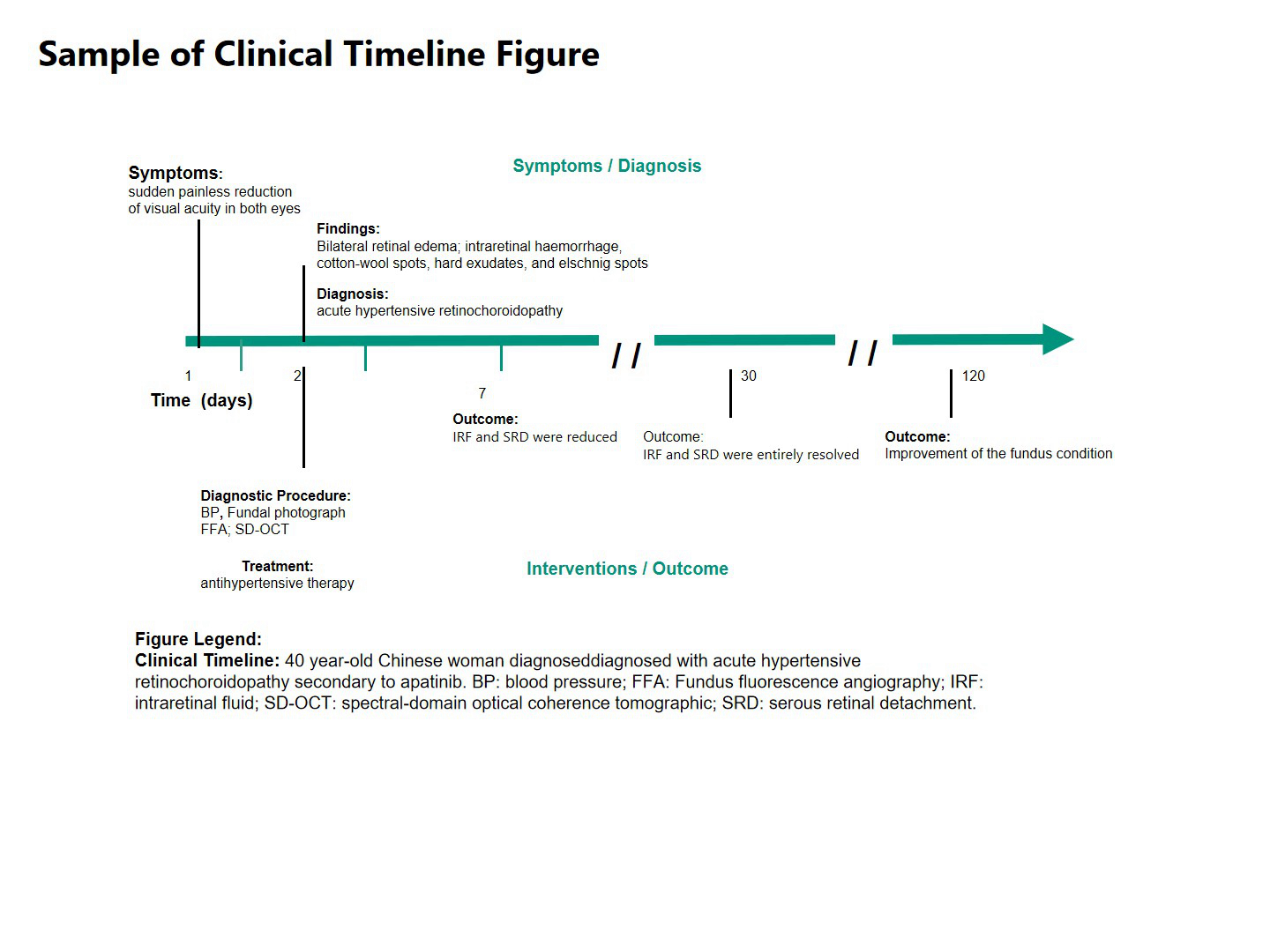

Supplement: Supplementary file 1 [file Image_1.JPEG]
